# Decoding Plant and Animal Genome Plasticity from Differential Paleo-Evolutionary Patterns and Processes

**DOI:** 10.1093/gbe/evs066

**Published:** 2012-07-24

**Authors:** Florent Murat, Yves Van de Peer, Jérôme Salse

**Affiliations:** ^1^INRA/UBP UMR 1095 GDEC ‘Génétique, Diversité et Ecophysiologie des Céréales’, Clermont Ferrand, France; ^2^Department of Plant Systems Biology, VIB, Ghent, Belgium; ^3^Department of Plant Biotechnology and Bioinformatics, Ghent University, Ghent, Belgium

**Keywords:** synteny, duplication, evolution, genome, rearrangement, plasticity

## Abstract

Continuing advances in genome sequencing technologies and computational methods for comparative genomics currently allow inferring the evolutionary history of entire plant and animal genomes. Based on the comparison of the plant and animal genome paleohistory, major differences are unveiled in 1) evolutionary mechanisms (i.e*.,* polyploidization versus diploidization processes), 2) genome conservation (i.e*.,* coding versus noncoding sequence maintenance), and 3) modern genome architecture (i.e., genome organization including repeats expansion versus contraction phenomena). This article discusses how extant animal and plant genomes are the result of inherently different rates and modes of genome evolution resulting in relatively stable animal and much more dynamic and plastic plant genomes.

## Introduction

Genomes are the blueprints of all living organisms and underpin the mystery of life. Indeed, when considering the presence and absence of genes, expansions, or contractions of gene families, and topologies of *cis*-regulatory circuits, which in turn, might inform us on the importance of specific regulatory, metabolic, or developmental pathways, the genome sequence forms a tremendous resource providing fundamental insights into the functioning of an organism. Genomes thus represent the foundation from which many fundamental biological insights may be gained. Careful analysis of the genomic content and structure for founder and extinct ancestral karyotypes will further our understanding of the different genomic properties and how they came about in modern species. In particular, the deluge of genomic data, which has dramatically increased in recent years, now offers the opportunity to investigate, for the first time, and in a single analysis using the same methodological approach, paleo-evolutionary patterns and processes that have shaped present-day plant and animal genome organization.

Similarities and differences between plant and animal genome structure and evolution have long been a source of intense investigations, mainly based on few genomes comparisons or lacking a unified and transversal approach to perform comparative genomics in both kingdoms (Kejnovsky et al. 2009). In order to elucidate and understand the basic biological mechanisms that have shaped these genomes and associated key gene functions that emerged during the last 500 Myr of their evolution, we compared animal (vertebrates) and plant (monocots and dicots) genomes in a single study using the same methodological approach. This way, we unveil both common and specific evolutionary patterns and processes regarding 1) genome duplication events, 2) the evolution of gene families, 3) genome size and structure variation, and 4) repeat invasion and contraction. The reconstructed ancestral genomes thus represent a foundation that helps us in the current article to unravel the successive steps that contributed to their evolution and deciphering precisely why considering plant as dynamic and animal as more stable genomes.

## Materials and Methods

Genomes investigated in this study are presented in tables 1 and 2. Methods used for 1) genome comparisons, 2) reconstruction of ancestral karyotypes, and 3) investigation of gene/TE evolutionary trends are presented below. Because it is difficult to infer orthologous (derived from a common ancestor by speciation) and paralogous (derived by duplication within one genome) relationships from sequence comparisons, stringent alignment criteria and statistical validation are essential to evaluate accurately whether the association between two or more genes found in the same order on two chromosomal segments in different genomes occurs by chance or reflects true colinearity. When two genomic nucleotide/protein sequences are aligned, BLAST produces HSPs (high scoring pairs) that consist of two sequence fragments of arbitrary but equal length, which alignment is locally maximal and for which the alignment score meets or exceeds a threshold or cut-off score. HSPs are based on statistical criteria such as the *e* value, score, and percentage identity. However, the detection of conserved regions is limited when sequence alignments are obtained with these BLAST default parameters. To increase the significance of interspecific sequence alignments for inferring evolutionary relationships between genomes, we used parameters defined from BLAST results (Salse et al. 2009a). Plant and animal genomes (cf. tables 1 and 2) have then been compared in the current analysis through annotated CDS (for CoDing Sequences) alignments (using BLAST) using three parameters, that is, AL (aligned length = ∑ HSP lengths), CIP (cumulative identity percentage = ∑ nb ID by [HSP/AL] × 100) and CALP (cumulative aligned length percentage = AL/query length). The CIP corresponds to the cumulative percentage of sequence identity observed for all the HSPs divided by the cumulative AL, which corresponds to the sum of all HSP lengths. CALP is the sum of the HSP lengths (AL) for all HSPs divided by the length of the query sequence. With these parameters, it becomes possible to select the highest cumulative percentage identity over the longest cumulative length, thereby increasing stringency in defining uniquely conserved (orthologs) or duplicated (paralogs) gene pairs between two genome sequences. These two thresholds have been used to compare plant and animal genomes depending on their evolutionary relationships: CIP/CALP of 70% and 50% for genomes deriving from common ancestors dating back to <50 million years ago (Mya) (i.e*.*, closely related) and >50 Mya (i.e*.*, distantly related), respectively (Salse 2012). Most of the comparative genomics studies performed to date in plants and animals were done without applying statistical validation of the results and therefore, the significance of the relationships established in different studies is difficult to assess. In our study, we have systematically performed a statistical test after the BLAST comparison with the CIP/CALP parameters to validate nonrandom associations between groups of sequences. CloseUp provides a single representation of the colinearity by looking for less than perfect linear gene correspondence between chromosome segments (Hampson et al. 2005). It is based on the following parameters that relate to the gene density ratio, gene cluster length, and match number between orthologs. Our statistical validation is equivalent to a CloseUp analysis based on a density ratio of 2, a cluster length of 20, and a match number of 5.

**Table 1:** Plant/Animal Genome Data Sets Used in Paleogenomics Studies

Species	Common Name	Chromosomes	Genome (Mb)	Annotated Genes	Synteny	Duplication	WGD	References
Plants (monocots and dicots)								
*Oryza sativa*	Rice	12	372	41,046	RG	448-10-73	1R	IRGSP (2005)
*Sorghum bicolor*	Sorghum	10	659	34,008	6147-12-99	409-10-84	1R	Paterson et al. (2009)
*Zea mays*	Maize	10	2365	32,540	4454-30-82	3454-17-99	2R	Schnable et al. (2009)
*Brachypodium distachyon*	Brachypodium	5	271	27,601	8533-12-99	642-13-79	1R	IBI (2010)
*Vitis vinifera*	Grape	19	302	21,189	RG	543-23-71	1R	Jaillon et al. (2007)
*Arabidopsis thaliana*	Cress	5	119	33,198	2389-80-99	1630-55-83	3R	AGI (2000)
*Populus trichocarpa*	Poplar	19	294	30,260	4555-87-92	4164-46-73	2R	Tuskan et al. (2006)
*Glycine max*	Soybean	20	949	46,194	4013-164-97	9533-89-55	3R	Schmutz et al. (2010)
*Fragaria*	Strawberry	7	208	32,630	3289-94-70	114-27-19	1R	Shulaev et al. (2010)
*Theobroma cacao*	Cacao	10	218	27,814	4472-21-81	370-19-66	1R	Argout et al. (2011)
*Malus x domestica*	Apple	17	528	58,984	3498-104-70	2845-69-59	2R	Velasco et al. (2010)
Total					27135-695-81	19559-396-57		
Animals (vertebrates)								
*Homo sapiens*	Human	23	3059	18,794	RG	128-29-33	2R	IHGSC (2001)
*Mus musculus*	Mouse	20	2635	19,380	10088-143-75	48-21-13	2R	MGSC (2002)
*Canis familiaris*	Dog	39	2445	42,626	5551-97-71	76-26-19	2R	Lindblad-Toh et al. (2005)
*Equus caballus*	Horse	32	2360	18,838	10195-83-83	134-24-26	2R	Wade et al. (2009)
*Monodelphis domestica*	Oppossum	9	3502	31,265	3413-79-78	16-7-4	2R	Mikkelsen et al. (2007)
*Gallus gallus*	Chicken	33	1032	30,077	2311-41-91	41-17-25	2R	Wallis et al. (2004)
*Oryzias latipes*	Medaka	24	721	17,117	2124-238-60	830-125-39	3R	Kasahara et al. (2007)
*Pan troglodytes*	Chimpanzee	24	3175	40,460	5091-56-62	43-11-15	2R	CSAC (2005)
Total					38773-737-74	1316-260-22		

Note.—Data for number of annotated genes are taken from Phytozome (http://www.phytozome.net) and PLAZA (http://bioinformatics.psb.ugent.be/plaza). Synteny data includes number of orthologs, number of blocks, and percent of genome covered. Duplication data includes number of paralog, number of blocks, and percent of genome covered. Column eight (WGD, whole genome duplication) indicates the number of polyploidization events (R, rounds). RG, reference genome, indicating that rice (*Oryza sativa*), grape (*Vitis vinifera*), and human (*Homo sapiens*) have been used as reference genomes for the synteny analysis for the eudicots, monocots, and vertebrates, respectively.

**Table 2:** Major Differences in Plant and Animal Genome Structure, Function, and Evolution

Genome Properties	Features	Plants (monocots and dicots)	Animals (vertebrates)	References
Ancestor				
	Protochromosomes	5–7	10–12	CA
	Protogenes	∼10,000–15,000	∼13,000–20,000	CA
	Gene space size	∼25 Mb	∼50 Mb	CA
Structure				
	Chromosome/genomes	Shuffled	Stable	CA
	Genes (size, exon size, exon number)	2.9 Kb/384 bp/4.7	39.7 Kb/290 bp/8.5	CA
	CNS	Short/less conserved	Long/highly conserved	Reineke et al. (2011)
	Gene families	Less/genome wide	Numerous/tandem	Kejnovsky et al. (2009)
	TE	Mainly class I LTR / recent	Mainly class I non-LTR / old	CA
Function				
	Neo/sub-functionalization	High between duplicates	Low	Pont et al. (2011)
	Splice variant	Low	High	Taher et al. (2011)
	Small RNA	miRNA/target coevolution	miRNA emergence/new target	Axtell et al. (2011)
Evolution				
	Duplication/polyploidy	Frequent/recent	Rare/old	CA
	Fusion	Centromeric-based	Telomeric-based	CA
	Recombination	High/variable	Low/stable	Gaut et al. (2007)
Plants versus animals	Chromosomes and genomes	Plastic	Stable	CA

Note.—CA, current analysis; compared with those discussed from the literature (references cited).

Different approaches such as 1) cladistic (Dobigny et al. 2004), 2) GRIMM (genome rearrangements in man and mouse, Tesler 2002), 3) MGR (multiple genome rearrangement, Bourque et al. 2004), and (iv) DUPCAR (contiguous ancestral regions, Ma et al. 2008) have been developed to reconstruct ancestral genomes in vertebrates. However, because of the difference in evolutionary history and derived gene conservation rate in plant and animal (see Results), most of these methods cannot be transferred to plant paleogenomics purposes. In order to be able to use the same unique approach in the current analysis in both plant and animal lineages, the reconstruction of ancestral karyotypes is obtained by computing common intervals of conserved blocks between two genomes (i.e*.,* derived from the validated orthologous genes/blocks) and within a single genome (i.e*.*, derived from the validated paralogous genes/blocks) into contiguous ancestral regions (CAR). Chromosomal blocks that are duplicated in two different genomes but located at orthologous positions when comparing the two genomes are considered as 1) unique in the ancestor (i.e., CAR), and 2) deriving from a shared prespeciation duplication event. On the contrary, a chromosomal block that is duplicated in one genome but not identified as duplicated at orthologous positions when comparing two genomes is considered as 1) a species-specific duplication, and 2) deriving from a postspeciation duplication event. The same approach is applied for any type of rearrangements including inversions and translocations. From the identified CARs, the most likely evolutionary scenario is proposed on the following assumptions: 1) ancestor modeling is based on duplications (or any shuffling events) found at orthologous positions between modern species, and thus considered as ancestral, 2) evolutionary history is based on the smallest number of shuffling operations (including inversions, deletions, fusions, fissions, translocations) that explain evolution from the ancestral genome to modern karyotypes. Based on these assumptions, the most likely ancestral karyotypes and associated evolutionary scenarios have been proposed for plants and animals.

Finally, the comparison of gene/TE evolutionary dynamics in plants and animals has been investigated based on the genome annotation features published previously (see references in tables 1 and 2). Annotated genes and TEs were considered within orthologous, paralogous blocks, as well as CARs defined previously. Distribution curves were then constructed in collinear/duplicated regions with CDS, TE class I and class II annotation features.

## Results and Discussion

### Plant Genome Paleohistory

More than 30 land plant (more than 20 of which are flowering plants) genome sequences are available either as chromosome anchored sequences, unmapped or partial whole-genome sequences, and if not sequenced, at least associated with high-resolution gene-based genetic maps, all allowing evolutionary comparative genomics studies at unprecedented resolution (cf. table 1 presenting 11 pseudomolecule-based plant genome sequences available at Phytozome [http://www.phytozome.net; Goodstein et al. 2012] and PLAZA [http://bioinformatics.psb.ugent.be/plaza; Van Bel et al. 2011]). Paleogenomics, or the reconstruction of the ancestral genome structure of modern species, is based on large-scale comparative genomic analyses to identify shared- and lineage-specific shuffling events, prior founder karyotype reconstruction. We used specific parameters to compare genomes, reconstruct ancestral karyotypes, and decipher genes/TEs evolutionary trends (see Materials and Methods).

Comparison of 11 plant genomes that diverged from a common ancestor 150–300 Mya unraveled 27,135 orthologous gene relationships defining 685 collinear blocks and covering on average 81% of the considered plant genomes, cf. table 1. Our data support that between 10% and 20% (for >50 Mya of divergence) up to 60%–80% (for <20 Mya of divergence) of the genes are conserved as strict orthologs when considering sequence conservation based on sequence alignment parameters, as well as gene order/orientation maintenance (table 1). This analysis complements and largely refines previous analyses performed on a much smaller number of genomes (Abrouk et al. 2010) or done separately within monocots and dicots (Salse et al. 2008, 2009b; Murat et al. 2010). Using the approach described previously, every single considered plant genome, although diploid in their current structure, harbors duplicated genes (19,559 gene pairs in total) defining paralogous segments (396 duplicated blocks in total) that cover on average 57% of the genome space (table 1). Integration of intraspecies duplication and interspecies synteny analyses allowed precise confirmation of seven shared ancestral duplications recovered in all plant genomes investigated, covering >50% of any considered genome in eudicots and monocots, and provides clear proof of whole genome duplication (WGD, also referenced to hereafter as R for rounds of duplications) events, demonstrating that these diploid plant species are all diploidized ancient polyploids (Van de Peer, Fawcett et al. 2009; Van de Peer, Maere et al. 2009; Tang et al. 2010; Jiao et al. 2011). Our data refined previous analyses of plant genome conservation and duplication patterns (Abrouk et al. 2010; Murat et al. 2010; Proost et al. 2011) based here on the largest number of genome sequences that allowed the delineation of an ancestral plant karyotype (APK) with a minimal physical size (i.e*.*, cumulative conserved CDS size) of ∼25 Mb, structured in five protochromosomes in monocots (with an alternative *n* = 7 structure proposed in Salse 2012) or seven (in eudicots) protochromosomes, and comprising a minimum of 10,000–15,000 protogenes (table 2).

**Fig. 1.—:**
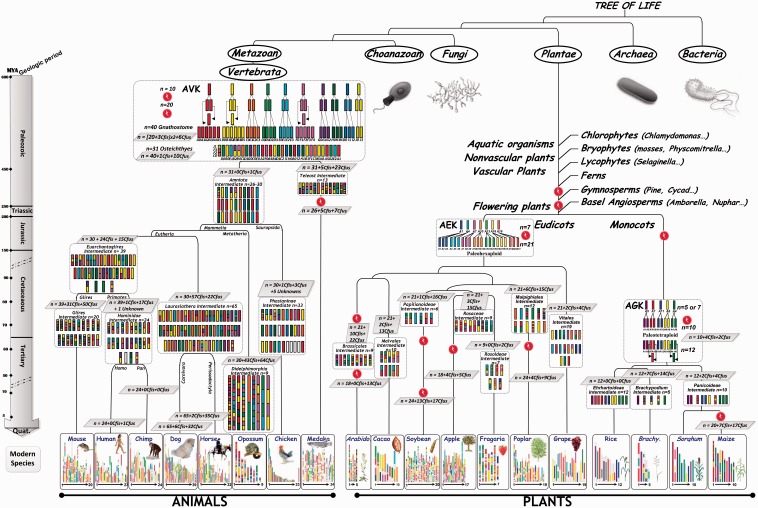
Evolutionary scenario of plant and animal genomes. Plant (right) and animal (left) chromosomes are represented with color codes to illustrate the evolution of segments from their founder ancestors (top) with 7 (eudicots, referenced as AEK for ancestral eudicot karyotype), 5/7 (monocots, referenced as AGK for ancestral grass karyotype), and 10/12 (vertebrate-based metazoans, referenced as AVK for ancestral vertebrate karyotype) protochromosomes. The WGD events that have shaped the structure of the different plant and animal genomes during their evolution from their common ancestors are indicated as red dots along with the geologic periods (Jurassic, Cretaceaous, Paleogene, and Neogene) and corresponding time scale (in Mya) at the left. The present-day structure of the 19 plant and animal genomes are shown at the bottom of the figure. Evolutionary events such as chromosome fissions (Cfis) and fusions (Cfus) are mentioned in gray boxes, as well as ancestral intermediate chromosome numbers (*n*).

The refined characterization of seven paleoduplications and the inference of the relationships between different conserved regions allowed re-evaluating the evolutionary events that have shaped the monocot genomes since their divergence from a putative ancestor with five chromosomes. About 50–90 Mya, the *n* = 5 ancestor (AGK, ancestral grass karyotype) went through a WGD (1R ancestral), followed by four chromosome fissions (hereafter Cfis) and two fusions (hereafter Cfus) that resulted in an *n* = 12 ancestral intermediate (fig. 1, right). An alternative scenario involving a *n* = 7 ancestor tetraploidized into a *n* = 14 intermediate followed by two protochromosomal fusions delivering the *n* = 12 founder monocot ancestor has been proposed (Salse 2012). We suggest that the monocot genomes derived from this intermediate consequently evolved through the following events: 1) rice retained the original chromosome number of 12, 2) the maize and sorghum genomes evolved through two Cfis and four Cfus that resulted in a *Panicoideae* ancestor with *n* = 10 chromosomes, and 3) *Brachypodium* evolved through 7 Cfis and 14 Cfus that resulted in a basic number of *n* = 5 chromosomes. Furthermore, we proposed that the maize genome underwent a recent specific WGD (thus 2R in total) event, resulting in an intermediate with *n* = 20 chromosomes, followed rapidly by at least 17 Cfus and 7 Cfis leading to the modern genome structure with 10 chromosomes.

Similarly, the identification of remnants of an hexaploidy event (i.e*.,* seven triplicated blocks, all triplicates dating back to ∼200 Mya) in all the eudicot genomes analyzed favors the model with a hexaploid ancestor (seven protochromosomes followed by an ancestral 1R), with an *n* = 21 intermediate (AEK for ancestral eudicot karyotype) common to all eudicots. In this scenario, the grape, fragaria, and cacao genomes all evolved from this intermediate through respectively 2Cfis–4Cfus, 3Cfis*–*17Cfus, and 2Cfis*–*13Cfus, to reach their modern genome structure. The poplar and apple genomes subsequently underwent specific WGD (2R in total) events. The poplar genome structure derived from a *Malpighiales* intermediate of 12 chromosomes (12 = 21+6Cfis+15Cfus) that have been duplicated (*n* = 24) with then 4Cfis*–*9Cfus to reach its *n* = 19 modern genome structure. The apple genome structure derived from a *Rosaceae* intermediate of nine chromosomes (9 = 21+3Cfis+15Cfus) that has been duplicated with then 4Cfis*–*5Cfus to reach its *n* = 17 modern genome structure. Finally, we propose that the *Arabidopsis* and soybean genomes evolved from an *n* = 21 intermediate through two specific WGD (3R in total). The *Arabidopsis thaliana* genome structure was derived from a *Brassicales* intermediate of nine chromosomes (9 = 21+10Cfis+22Cfus) that has been duplicated (*n* = 18) with then 13Cfus to reach its *n* = 5 modern genome structure. Finally, we suggest that the soybean genome structure derived from a *Papilionoideae* intermediate of six chromosomes (6 = 21+1Cfis+16Cfus) that have been duplicated (*n* = 24) with then 13Cfis*–*17Cfus to reach its *n* = 20 extant genome structure, cf. figure 1, centre. Figure 1 illustrates that plant chromosome number reductions were the result of recurrent series of ancestral chromosome fusions due to both centromeric chromosome fusion (CCF) events (i.e., insertions of a chromosome into the centromeric region of another chromosome) or telomeric chromosome fusion (TCF) (i.e., fusion of two independent ancestral chromosomes by the telomere). CCF has been reported in the literature as NCF for nested chromosome fusion (Murat et al. 2010) so that 1NCF = 1Cfis*+*2Cfus.

Our previously obtained data and conclusions regarding the WGD pattern in monocots and dicots can be discussed regarding the mass species extinction events reported in the literature. Based on dating, the shared ancestral, or specific recent genome-wide duplication events, it has been proposed that paleopolyploidy events, usually considered as a rare and evolutionary dead-end phenomenon, may have been the basis for species diversification and survival during mass species extinction periods (Fawcett et al. 2009; Van de Peer, Fawcett et al. 2009; Van de Peer, Maere et al. 2009; Salse 2012). Dating of such duplication events clearly identifies distinct types of WGD during plant evolution: ancestral WGD, family- or lineage-specific WGD. Although still debated, it has been previously suggested that the ancient paleopolyploidization events that occurred in monocot (50–70 Mya) could be associated with the Cretaceous/Paleogene (called K-PgT, 65 Mya) extinction (Fawcett et al. 2009). We propose here that other “waves” of genome duplication events could also be linked to periods of extinction such as the Triassic/Jurassic (called Tr–J, 200 Mya regarding the pelohexaploidization characterized in eudicots) transition or more recent periods during the Paleogene and Neogene (for the characterized family- or lineage-specific duplications). These latter recent time periods may correspond to the reported accelerated diversification 30–20 Mya, suggested based on the observed historical changes in the distribution of dry forest communities and biomasses (Becerra 2005; Couvreur et al. 2010). Additional and even older WGD have recently been reported in seed and land plants (Jiao et al. 2011; fig. 1). However, for these old events, chromosome–chromosome synteny relationships have eroded to the extent that ancestral karyotypes can no longer be proposed or related yet to more ancient mass species extinction events, then not investigated in the current analysis.

### Animal Genomes Paleohistory

Using the same methodological approach, comparison of eight metazoan (more specifically vertebrate) genomes (available from http://genome.ucsc.edu/) that diverged from a common ancestor >450 Mya allowed us to characterize 38,773 orthologous genes defining 737 collinear blocks and covering on average 74% of the considered genomes, showing that between 40% and 50% (for >50 Mya of divergence) and 60–80% (for <20 Mya of divergence) of the genes are conserved as strict orthologs between vertebrate genomes (table 1).

Our approach allowed us to refine and extend identification of interchromosomal duplications in vertebrate, providing a set of conserved duplicated genes (1,316 gene pairs in total) defining 260 paralogous blocks and covering on average 22% of any of the eight animal genomes considered (table 1). Integration of intraspecies duplication and interspecies synteny analyses allowed the characterization of 10 shared ancestral duplications (identified as double syntenic blocks) covering up to 39% (e.g., medaka) of the considered animal genomes, and providing clear proof of ancestral WGD events, demonstrating that modern animal species are all diploidized ancient polyploids (Vandepoele et al. 2004; Dehal and Boore 2005; Nakatani et al. 2007; Van de Peer, Fawcett et al. 2009; Van de Peer, Maere et al. 2009). The characterization of paleoduplications (i.e., inferred as double conserved syntenies) and the precise relationships between different conserved regions allowed us to identify evolutionary events that have shaped the modern animal genomes since their divergence from a putative ancestral vertebrate karyotype (AVK) (fig. 1) consisting of probably 10 protochromosomes (favoring Nakatani et al. 2007 scenario from our reconstructed *n* = 31 *Osteichthyes* ancestor described below), containing ∼13,000–20,000 protogenes covering a physical gene space size of ∼50 Mb, then complementing alternative inferences of the animal paleohistory (Panopoulou et al. 2003; Dehal and Boore 2005), as well as vertebrate protochromosomic structure of *n* = 10 (Nakatani et al. 2007), *n* = 11 (Kohn et al. 2006) and *n* = 12 (Jaillon et al. 2004) (table 2).

About 450 Mya, the *n* = 10 AVK went through a WGD (1R ancestral), followed by three interchromosomal translocations and fusions that resulted in an *n* = 23 ancestral intermediate (10+10+3 = 23 chromosomes). The *n* = 23 ancestor went through another WGD (2R ancestral; Van de Peer et al. 2010) shortly after the first WGD (Furlong and Holland 2002), followed by six chromosomal fusions to reach an *n* = 40 *Gnathostome* ancestor intermediate (23 × 2 − 6Cfus = 40 chromosomes). The modern animal genomes derived from a vertebrate common ancestor (*Osteichthyes*) of 31 protochromosomes resulting from 10Cfus and 1Cfis of the *n* = 40 *Gnathostome* intermediate. We propose that a teleost ancestor with 13 protochromosomes derived from the *n* = 31 vertebrate ancestor intermediate with 23Cfus and 5Cfis. The medaka genome may then have been derived from this *n* = 13 teleost ancestor including a specific WGD (referenced as ancestral 3R) and additional Cfus (7) and Cfis (5) events. Further, we propose that the chicken, opossum, horse, dog, chimp, human, and mouse genomes have been derived from an *Amniote* ancestor with 26–30 chromosomes derived from the *n* = 31 vertebrate ancestor intermediate followed by at least one Cfus. The chicken and opossum genomes derived directly from the *n* = 30 *Amniote* clade ancestor with respectively, 1Cfis–3Cfus and 43Cfis–64Cfus to reach their modern *n* = 33 chromosome and *n* = 9 chromosome present-day genome structures, respectively. Horse and dog derived from a *Laurasiathera* intermediate that supposedly had 65 protochromosomes obtained from the *n* = 30 *Amniote* clade ancestor plus 57Cfis–22Cfus. The modern horse and dog karyotypes were likely derived from the *n* = 65 *Laurasiathera* clade ancestor with respectively, 2Cfis–35Cfus and 6Cfis–32Cfus to reach their modern *n* = 32 chromosome and *n* = 39 chromosome present-day genome structures, respectively. Finally, human, chimp, and mouse derived from a common *Euarchontoglires* intermediate of 39 protochromosomes that have been shaped from the *n* = 30 *Amniote* clade ancestor with 24Cfis–15Cfus. The mouse genome structure corresponds to the one of the *n* = 39 *Euarchontoglires* intermediate including additional specific Cfus (50) and Cfis (31) events. Chimp and human differ only by a unique chromosome fusion event from their common *Hominidae* ancestor of 24 chromosomes that has been derived from the *n* = 39 *Euarchontoglires* intermediate ancestor with 1Cfis / (17 for chimp and 18 for human) Cfus. Figure 1 clearly illustrates that ancestral animal chromosomes fused by a TCF, “end-to-end” or “tip-to-tip” joining process. It has been suggested that such TCF events are mediated by repeat sequences in animals (Carbone et al. 2006).

Our previous data and associated conclusions that largely refine the public animal paleogenomics data (detailed previously) can now be considered in the context of published studies that discussed consequent impact sexual chromosomes on the reported WDG pattern. Overall, dating of the shared ancestral or specific recent duplication events identifies two distinct types of WGD (red dots in fig. 1) during animal paleohistory: ancestral shared 2R WGD (>450 Mya), lineage-specific R WGD (300–350 Mya specific to teleosts including the medaka genome) (Vandepoele et al. 2004, Friedman and Hughes 2011). Whereas, we have identified more recent lineage-specific WGDs in plants, none of such recent events has been identified in recent vertebrate history. Our current analysis refines the previous analyses from Jaillon et al*.* (2004) and Kohn et al*.* (2006) providing an integrated view of vertebrate paleogenomics with an ancestor of 10–12 protochromosomes followed by two rounds (2R) of WGDs leading to a *Gnathostome* ancestor of *n* = 40 chromosomes. We can speculate that additional WGDs have not been possible in animals once sexual chromosomes have been genetically determined, except for instance in invertebrates, fishes, and amphibians (fig. 1). The epigenetically mediated differentiation X/Y or Z/W chromosome systems early during animal evolution may render WGD deleterious because of impossible gamete reduction of polyploidy in this context. While in animals increased cytosine methylation of an ancestral Y (or W) chromosome provides the machinery to drive Muller’s ratchet making it as a nonrecombining and shorter chromosome, compared with its X (or Z) homoeolog, plant genomes immune from Muller’s ratchet evolved from homomorphic sexual chromosomes (Jamilena et al. 2008). This may explain the observed differences in recent lineage-specific WGD patterns between plants and animals. It could be argued that gene functional novelties derived from polyploidization may reduce the risk of plant species extinction (Fawcett et al. 2009; Van de Peer, Fawcett et al. 2009; Van de Peer, Maere et al. 2009), as has been suggested in mammals where vertebrate lineage extinction has been reported to have been higher in the preduplication paleohistory, that is, before the 2R events (Crow and Wagner 2006). This contrasted mode of evolution, illustrated in the figure 1 with few rearrangements of large chromosome segments in animal evolution scenario, explains the observed diversity between plant and animal karyotypes in terms of chromosome number and genome size as detailed in the next sections.

### Paleohistorical Consequences on Modern Genome Architectures

**Fig. 2.—:**
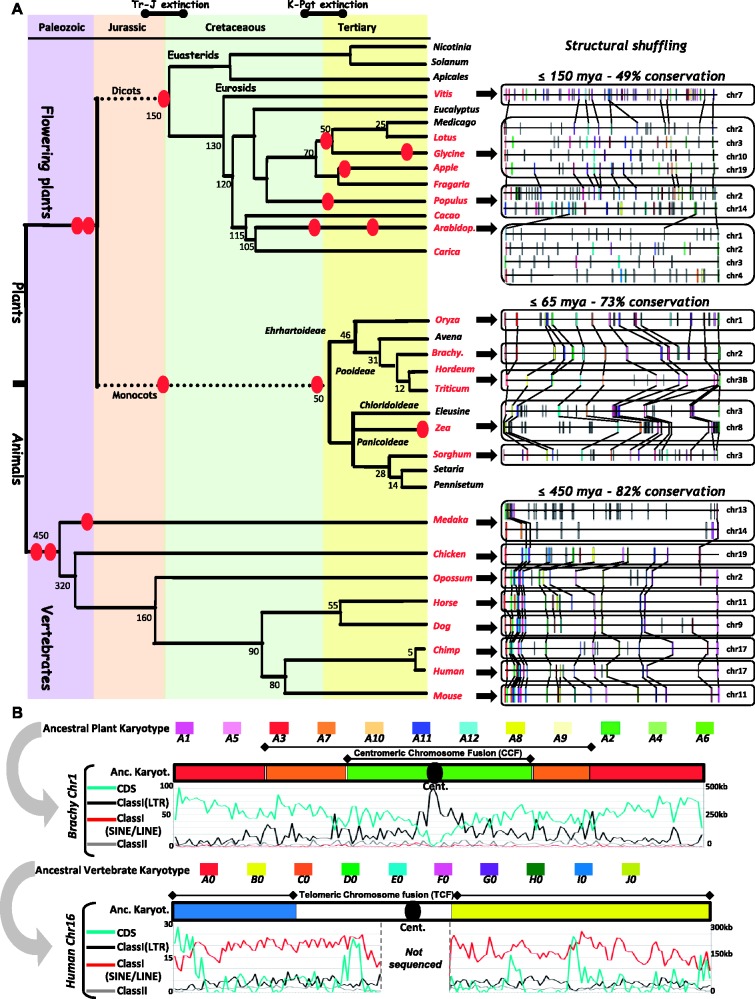
Gene conservation in plant and animal genomes. (*A*) A highly pruned phylogenetic tree of the plants and animals is shown at the left end side of the figure with speciation dates of the branches (in millions years) and duplication events highlighted as red dots. Micro-synteny conservation is shown at the right end side of the figure with homologous genes in the same color code and connected with black lines. (*B*) Comparison of plant (*Brachypodium* chromosome 1) and animal (human chromosome 16) genome heat maps. Each chromosome structure is illustrated based on the ancestral karyotype (10 and 12 colour codes, respectively for animals and plants) and associated with corresponding CDS (blue), TE class I LTR (black), TE class I non-LTR (purple) and TE class II (gray) distribution curves. Within 500-kb-sized windows covering the entire considered chromosome, CDS distribution (left) represents the number of annotated genes and TEs distribution (right, Y-axis) represents the cumulative size in “Kb” covered by either class I (black curve) and class II (gray curve) elements.

The previously obtained paleogenomics data (i.e., synteny/duplication detection, ancestral karyotype reconstruction, WGD characterization), based on the same analysis framework for both plant and animal genomes, allowed us to investigate the overall genome features that are similar and different in both lineages, at both the genic (current paragraph) and repeat (next paragraphs) levels. We show that within 20 Myr of separation, 10%–20% and 40%–50% of protein-coding genes have been conserved in plants and animals, respectively (tables 1 and 2; and illustrated in fig. 2*A*, right). We can suggest that the distinct rates of gene order conservation between plants and animals is the consequence of their different evolutionary patterns (fig. 1). While the metazoans (vertebrates in the current analysis) experienced no, or few rounds of WGDs because they have been derived from a *Gnathostome* common ancestor that had 40 protochromosomes (resulting from a paleo-octoploidized *n* = 10 ancestor), angiosperms experienced numerous WGDs from their common paleotetraploid of 12 protochromosomes (resulting from *n* = 5 of 7 ancestors) or paleohexaploid of 21 protochromosomes (resulting from a *n* = 7 ancestor) founder genomes, for the monocots and eudicots, respectively. This different rate of gene conservation may be directly linked to massive duplicated gene loss following WGD (Woodhouse et al. 2010; Schnable et al. 2011; Salse 2012) so that the genome colinearity in plants is shown as more eroded than that of animals over similar periods of time. Although polyploidy is rare in mammals, is has occurred in the speciation of many groups of fish (exemplified in the fig. 1 for medaka) and occurred also in amphibians and reptiles paleohistory (Le Comber and Smith 2004). Figure 2*A* illustrates such observed distinct pattern of gene colinearity retention in animal and plants with ∼70%–80% of gene conservation observed after an assumed 65 Myr of evolution in monocots compared with ∼450 Myr in animals. Whereas microcolinearity has been eroded by deletions and inversions between plant genomes that have diverged <100 Mya, microcolinearity between animal genomes can still be detected for much older divergences. However, despite WGDs, discussed previously, other factors such as generation or mating times and processes (e.g., vegetative multiplication in plants), genetic bottlenecks via natural selection, metabolic rates, and demography, cannot be excluded to explain such observed difference in genome content conservation between plants and animals (i.e*.,* mammals or vertebrates) (Donoghue and Purnell 2005).

We then estimate that, due to their distinct evolutionary histories, modern animal genomes are twice as conserved as plants regarding their gene content compared with plants. Our previous data and associated conclusion regarding differential rate of gene retention showing more evolutionary stability for animal than plant genomes, can directly explain previously published studies that established consequent differences between plant and animal genomes in 1) gene families structure, 2) recombination rate, 3) splicing variants, 4) conserved noncoding sequences, and 5) miRNA retention. The differential pattern of WGD reported here in both lineages has lead to the reported larger multigene families in plant (i.e*.*, >35% of *Arabidopsis* genes in families) compared with animals (i.e*.,* ∼1% of human genes in families) (Kejnovsky et al. 2009). Such described differences in gene family structures, with greater number of paralog in plants, may directly explain the observed difference in recombination rate. The higher recombination (homolog and nonhomolog) in plants, enhanced by duplicated blocks acting as substrate for ectopic recombination, contributed to a more dynamic and fluid genome structure (Gaut et al. 2007). The reported contrasted genome-wide conservation supports the identification in animals of, beyond the coding sequences, intergenic regions including gene promoter regions that lie in large conserved blocks, even comparing genomes that have diverged hundreds of millions of years ago (Wang et al. 2009). Overall, as the product of major differences in genome conservation, CNSs (conserved noncoding sequences) have been reported in mammals as larger (i.e., 69 to >100 bp) compared with small and highly degenerated in plants (i.e., >20 to 30 bp), (Lockton and Gaut 2005; Elgar and Vavouri 2008; Reineke et al. 2011). Moreover, as it is an often-observed phenomenon that gene duplication is more prevalent in plants, whereas on the contrary, alternative splicing might be more prevalent in animals (Lareau et al. 2004), we propose to consider as different mode of functional/expressional plasticity gained by the two lineages as a consequence of their differential paleo-evolutionary patterns and processes described in the previous sections. While gene shuffling following duplication in plants (through massive deletions or neo/subfunctionalization of duplicates, Pont et al. 2011; Adams and Wendel 2005) drives genome remodeling, animals have more splice variants to add variation to the proteome with a large proportion of human ones associated to genetic disorders (Barash et al. 2010). In terms of distinct evolutionary trends, plant genomes changed more quickly with more duplicates where animals developed more splice variants of conserved regulatory network (Taher et al. 2011). The reported distinct processes of evolution also explain the observed differences in miRNA biogenesis and functions in plants and animals (Axtell et al. 2011; Abrouk et al. 2012; table 2). Evolution of angiosperms through a series of WGDs explains the specific interactions (i.e., sequence similarities) between miRNA and their associated targets resulting in a more focused and localized effect to avoid misinteraction with numerous paralogs derived from paleoduplications (Brodersen et al. 2008; Abrouk et al. 2012). At the opposite, we can relate the low complexity of MIRNA/target interaction where single miRNAs can influence a broad set of genes to the stability of the mammal genomes, in order to overpass or at least attenuate it (Brodersen et al. 2008). Despite difference in miRNA function, the distinct mode of emergence is also impacted by the evolutionary process. The miRNA birth model in plants involves hairpin-based genic duplications, whereas in mammals it is assume the newborn miRNA derive from RNA hairpins (Axtell et al. 2011). We can then speculate that miRNA emergence and function is the result of the different mode of genome evolution in plants and animals. Overall, as a result of distinct paleohistorical scenarios unraveled in this study, gene distribution is different between modern plant and animal genomes. Figure 2*B* (blue curve) illustrates the gene distribution observed for *Brachypodium* chromosome 1 and human chromosome 16, where gene density increases on plant chromosomes from pericentromeric regions to subtelomeric regions, in human, genes appear in large gene islands (blue peaks), which are absent in plants.

### Paleohistorical Consequences on Repeat Mobility

Similar to genes, also transposable elements (TE) can be investigated based on the previously proposed paleogenomics data between the two major eukaryotic lineages to study their differential contribution to genome invasion and contraction. Transposable elements are ubiquitous in eukaryotes (consequently investigated in both plants and animals in the current analysis) and are typically divided into two classes (Wicker et al. 2007). Class I is represented by the retrotransposons (long interspersed nucleotide elements [LINEs], short interspersed nucleotide elements [SINEs], long terminal repeats [LTRs], and endogenous retrovirus [ERVs]). According to the modern classification, LTR retrotransposons are divided into two superfamilies: Copia (Pseudoviridae) and Gypsy (Metaviridae). Class II TEs, or DNA transposons, utilize DNA-based modes of transposition including “cut-and-paste” mechanism, rolling-circle replication, and a mechanism that involves DNA polymerase and is not yet well understood. Currently, 10 superfamilies of Class II DNA transposons are recognized in eukaryotes (Feschotte and Pritham 2007). The comparative genomics analysis performed using the same methodological approach as applied for true protein-coding genes (see previous section) allows to investigate differences in TE content and distribution. About 45% of the human genome is derived from transposable element sequences, whereas other genomes, especially those of plants, may consist of substantially higher proportions (up to 80% for the Triticeae) of transposable element-derived DNA (tables 1 and 2). Besides differences in TE content, also biases in TE distribution can be observed in animals and plants. Figure 2*B* illustrates the TE distribution (class I LTR as black curves, class I non-LTR LINE/SINE as purple curves, and class II as gray curves) observed for *Brachypodium* chromosome 1 and human chromosome 16. Where TE density is homogenous for human (on average, 9% and 33% of 500 Kb windows covered by respectively, class I LTR, as well as LINE/SINE), in plants the nested mode of insertion and reduction by illegitimate recombination produced hot spots of TEs (up to 58% of 500 Kb windows covered by class I TE) separated by low density TE regions (on average, 23% of 500 Kb windows covered by class I TE). Although class II elements are widespread and active in a variety of eukaryotes, they have been thought to be transpositionally inactive with, no, or few signs of recent activity in mammalian genomes, that is, 3% class II in human compared with plant, 5% class II in *Brachypodium*.

The large majority (>55%) of the TEs recognizable in the human genome were inserted prior to the radiation of mammals, ∼80–100 Mya, with an exceptional burst of SINES 40 Mya to reach the present number of more than 1 million of mainly inactive copies (Deininger et al. 2003). Recent evidence indicates that among the non-LTR retrotransposons, only some long interspersed nucleotide elements-1 (LINE-1, covering up to 20% of mammalian genomes) and short interspersed element (SINE) (*Alu* is the most abundant repetitive element in the human genome) subfamilies continue to be mobile in mammals today (Mills et al. 2006, 2007). Whereas ancient and short (up to few Kb) class I TE elements characterize modern animal genomes, long (up to several 10 Kb) LTR retrotransposons have been especially successful colonizers of plant chromosomes (from 25% in *Brachypodium* up to 50% in cacao). Overall, ancient, as well as recent Class I LTR TE invasion with a nested mode of insertion so that ancient elements are removed by younger ones through LTR-based illegitimate recombination. Such classical pattern of recent invasion of plant genome may have led to large genome size variation at the interspecific or more interestingly intraspecific levels. Panaud and collaborators reported a 2-fold genome size variation between rice genotypes by the burst of three LTR–retrotransposon families consisting in the accumulation of more than 90,000 retrotransposon copies during the last 3 Myr (Piegu et al. 2006), resulting in the rice genome size having doubled during this period (Kumekawa et al. 1999). Overall, as a result of distinct patterns of TE dynamics, TE distribution (mainly class I TE) is different between modern plant and animal genomes, with LTR (*copia* and *gypsy*) and non-LTR (LINEs and SINEs) retrotransposons, respectively predominant in plant and animals.

In conclusion, comparative analyses reveal the mechanisms that give rise to the different structure and evolution of plant and animal genomes. Polyploidy, more frequent in plants than animals, may trigger genetic and epigenetic changes (Adams and Wendel 2005) of the genomes leading to constant genome restructuration and reprogramming. As a consequence of these different paleo-evolutionary patterns and processes, plant genomes appear much more dynamic and faster evolving, whereas mammals are more conserved and stable.
